# 2428. Association of body mass index and bloodstream infections in patients on extracorporeal membrane oxygenation: A single-center retrospective cohort study

**DOI:** 10.1093/ofid/ofad500.2047

**Published:** 2023-11-27

**Authors:** Eun Hwa Lee, Jung Ah Lee, Jin Young Ahn, Su Jin Jeong, Nam Su Ku, Jun Yong Choi, Joon-sup Yeom, Young Goo Song, Se Hee Park, Jung Ho Kim

**Affiliations:** Yonsei University College of Medicine, Seoul, Seoul-t'ukpyolsi, Republic of Korea; Yonsei University College of Medicine, Seoul, Seoul-t'ukpyolsi, Republic of Korea; Yonsei University College of Medicine, Seoul, Seoul-t'ukpyolsi, Republic of Korea; Yonsei University College of Medicine, Seoul, Seoul-t'ukpyolsi, Republic of Korea; Division of Infectious Diseases, Department of Internal Medicine, Yonsei University College of Medicine, Seoul, Seoul-t'ukpyolsi, Republic of Korea; Yonsei University College of Medicine, Seoul, Seoul-t'ukpyolsi, Republic of Korea; Division of Infectious Diseases, Department of Internal Medicine, Yonsei University College of Medicine, Seoul, Seoul-t'ukpyolsi, Republic of Korea; Yonsei University College of Medicine, Seoul, Seoul-t'ukpyolsi, Republic of Korea; Chaum Life Center, CHA University, Seoul, Seoul-t'ukpyolsi, Republic of Korea; Yonsei University College of Medicine, Seoul, Seoul-t'ukpyolsi, Republic of Korea

## Abstract

**Background:**

Obesity is associated with poor clinical outcomes in critically ill patients. However, under some clinical conditions, obesity has protective effects. Bloodstream infections (BSI) are one of the most common nosocomial infections associated with extracorporeal membrane oxygenation (ECMO) support. BSI during ECMO is associated with higher mortality rates and poorer clinical outcomes. This study analyzed whether body mass index (BMI) was associated with BSI during ECMO and in-hospital mortality.

**Methods:**

All adult patients who had received ECMO support for >48 h were included in the analysis. The total duration of ECMO support, in-hospital mortality, and BSI were analyzed according to BMI categories. The Cox proportional hazards model was used to compare the risk of BSI according to different BMI categories.

**Figure 1.**
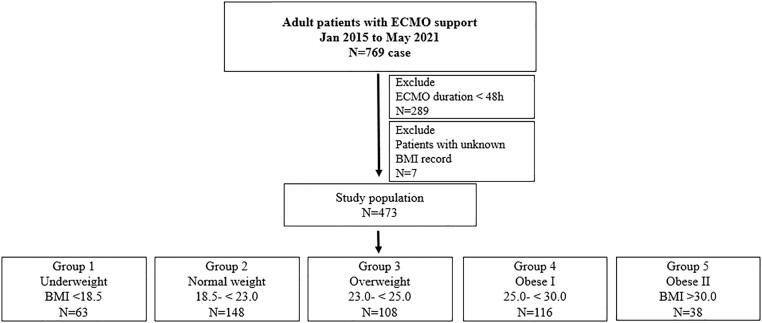
Study flow chart

**Results:**

A total of 473 patients were enrolled in the study. The average age was 56.5 years old and 65.3% were men. The total ECMO duration was approximately 11.8 days with a mortality rate of 47.1 %. The incidence of bacteremia and candidemia were 20.5% and 5.5%, respectively. There was no significant difference in bloodstream infection rates (p=0.784) or mortality rate (p=0.253) among the five groups. The underweight group required ECMO for respiratory support, whereas the overweight and obese groups required cardiogenic support (p< 0.0001). However, there was no significant difference in the development of BSI during ECMO support or in-hospital mortality among any of the five groups.

**Figure:**
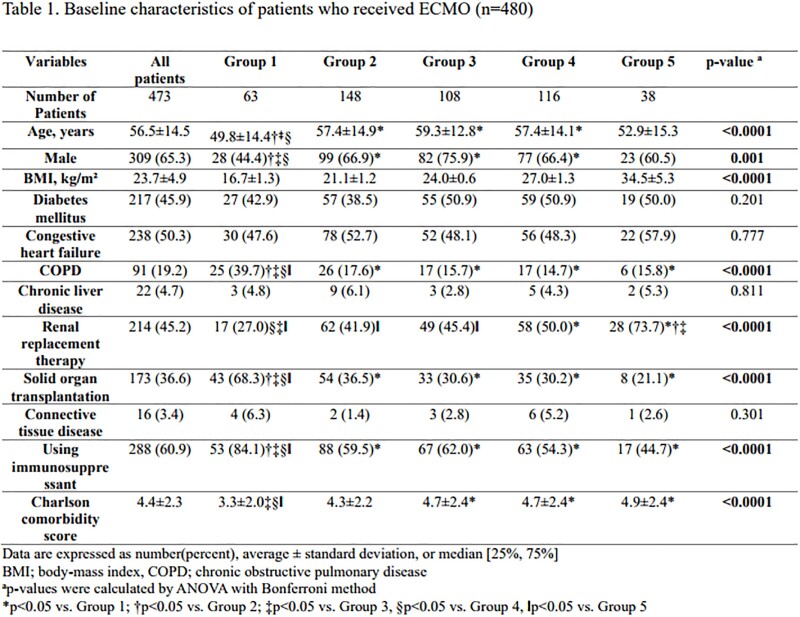

**Figure:**
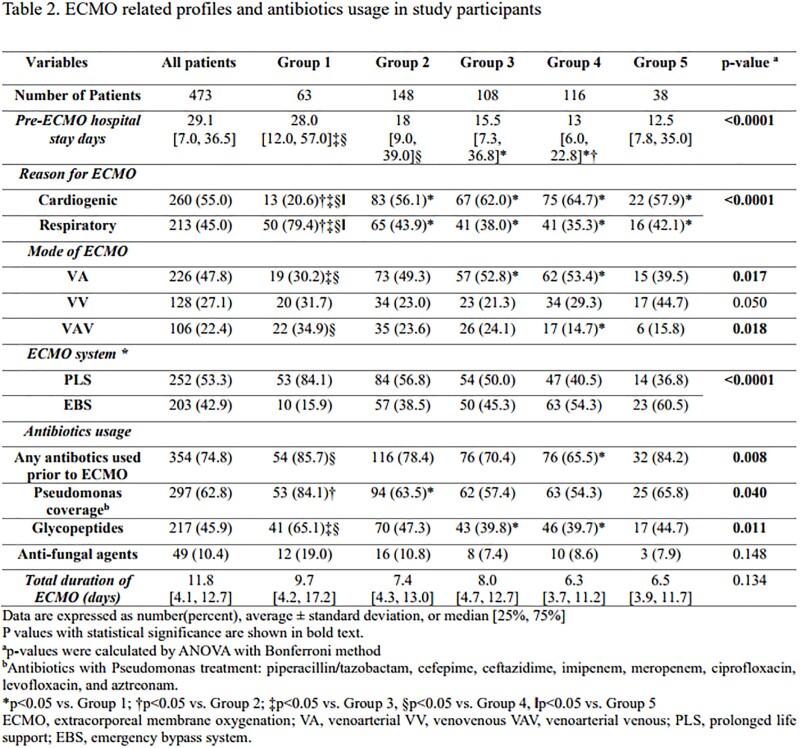

**Figure:**
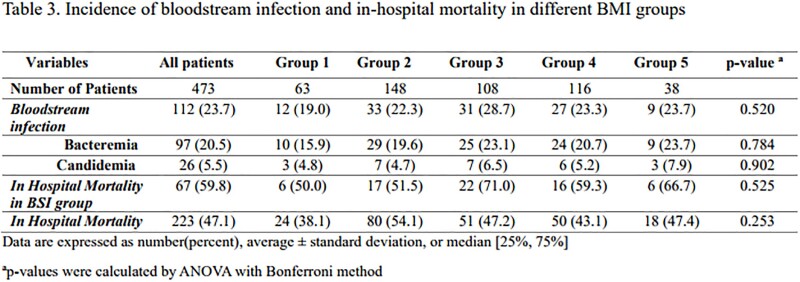

**Conclusion:**

Patients with higher or lower BMI did not show an increased risk of BSI or in-hospital mortality associated with ECMO support.

**Figure 2.**
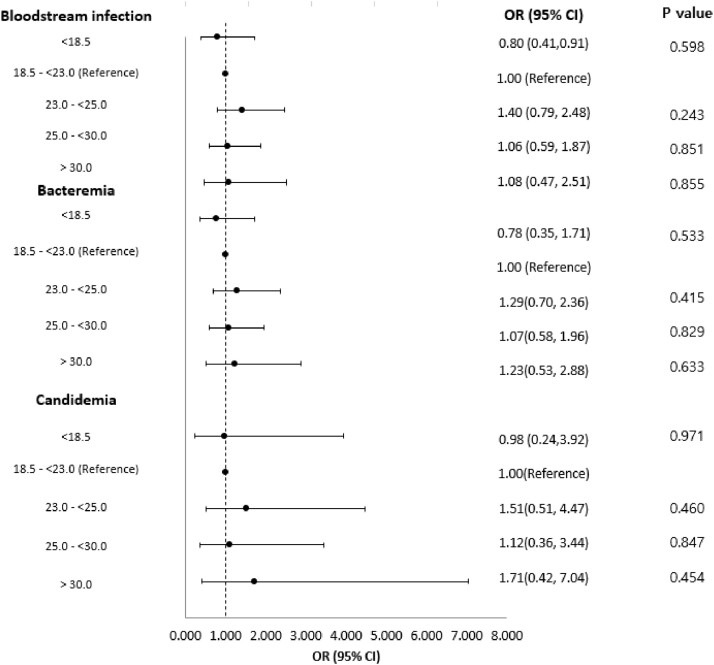
Forest-plot for risk of bloodstream infection occurrence in different body-mass index categories.

**Figure 3.**
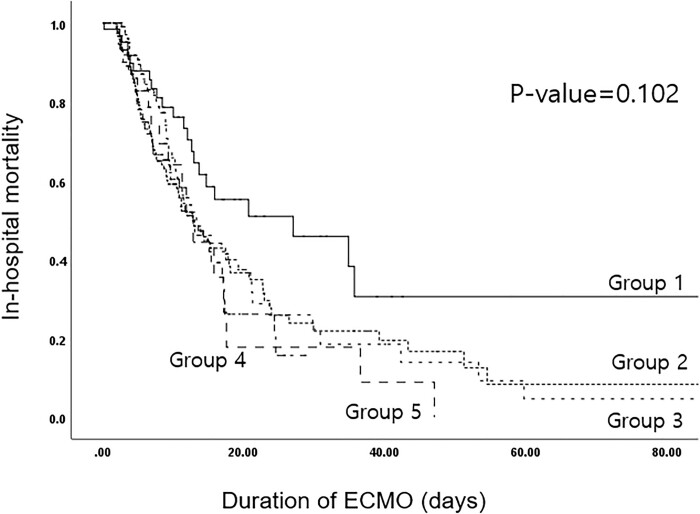
Kaplan-Meier analysis comparing the survival rate in different BMI groups.

**Disclosures:**

**All Authors**: No reported disclosures

